# The infraacetabular screw – anatomy, radiology, biomechanics and clinics

**DOI:** 10.1007/s00402-024-05528-7

**Published:** 2024-09-13

**Authors:** Axel Gänsslen, Jan Lindahl, Richard A. Lindtner, Dietmar Krappinger

**Affiliations:** 1 Trauma Department, Hannover Medical School, Hanover, Germany; 2 Department of Trauma and Orthopaedics, Johannes Wesling Hospital, Minden, Germany; 3 Department of Orthopaedics and Traumatology, Helsinki University Hospital and University of Helsinki, Helsinki, Finland; 4 Department of Orthopaedic and Trauma Surgery, Medical University Innsbruck, Anichstraße 35, Innsbruck, 6020 Austria

**Keywords:** Acetabular fracture, Infraacetabular screw, Anatomy, Radiology, Biomechanics

## Abstract

Acetabular fracture surgery follows the primary aim of anatomic reduction and rigid stable fixation of the fracture. Infraacetabular screws (IAS) allow for an increased stability of the acetabular fixation by closing the periacetabular fixation frame without requiring an additional posterior approach. The osseous screw corridor for infraacetabular screws use the transition zone between the acetabular ring and the obturator ring. The infraacetabular screw corridor (IAC) shows a double-cone shape with an isthmus located near the acetabular fovea. The iliopectineal eminence (IE) is mainly used as a clinical landmark for the intraoperative assessment of the entry point of IAS. The inlet view, the combined obturator oblique outlet view and a 1/3 iliac oblique outlet view may be used for the intraoperative radiological assessment for both the entry point and the screw trajectory of IAS. Several biomechanical studies have shown that IAS increase the stiffness of the internal fixation. Scientific proof for an improved clinical outcome is still missing.

## Anatomy

### Anatomy of the hip bone

**Fig. 1:**
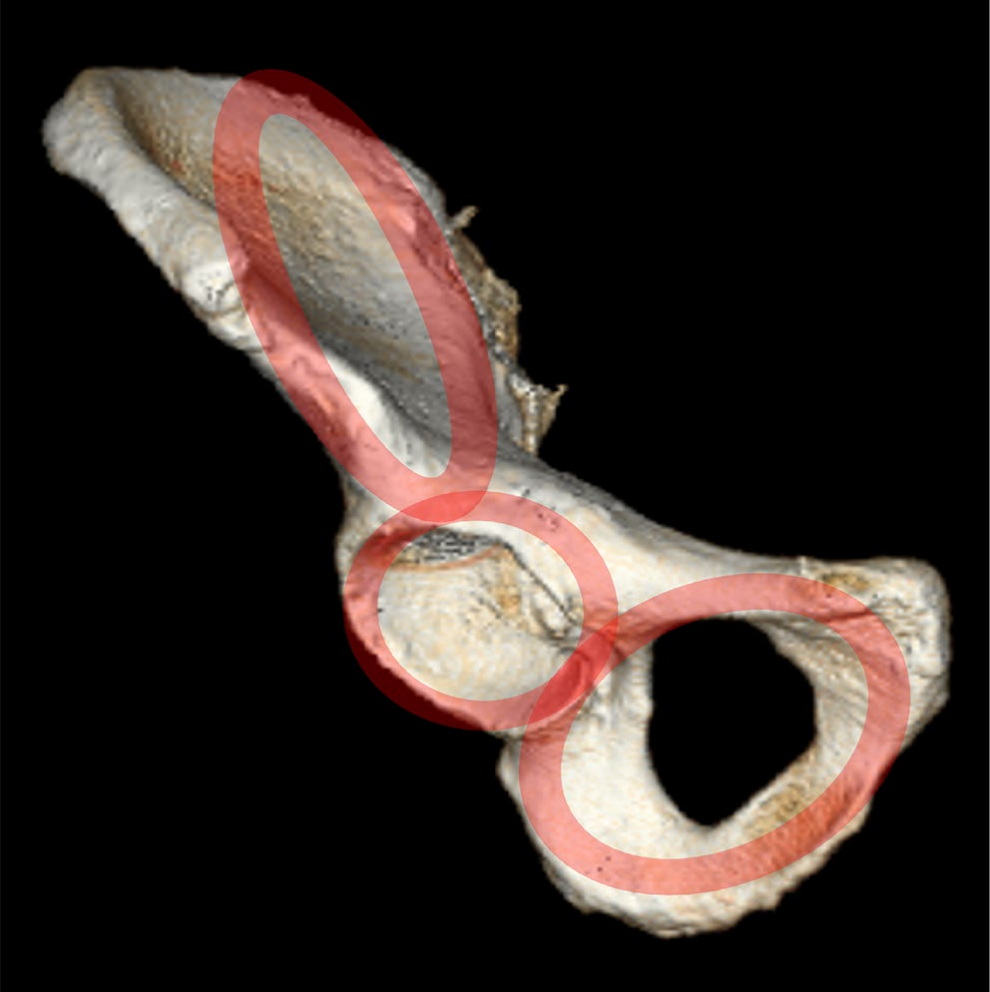
Three-ring structure of the hip bone consisting of an iliac wing ring, an acetabular ring and and obturator ring

From an anatomical point of view, the hip bone (os coxae) may be considered as a ring construction consisting of three rings: (a) an iliac wing ring, (b) an acetabular ring and (c) an obturator ring (Fig. 1 [1]), . The iliac wing ring consists of dense cortical bone with the iliac crest serving as its superior border. This ring surrounds a very thin central bone plate, which is interrupted by the gluteus medius pillar. In some patients an osseous void in this central bone plate posterior to the gluteus medius pillar is observed. Following Wolf´s law from 1870 [2], it is reasonable to assume that there will be an ubiquitous ring structure with a central void in the future of human mankind. The acetabular ring consists of the lunate surface with its surrounding bone, namely the anterior wall, the thicker posterior wall and the superior dome. The ground floor of the central acetabular fossa consists of bone with a thickness of 3–4 mm [3]. This second ring is internally rotated in relation to the iliac wing ring. The obturator ring is the actual ring structure revolving the obturator foramen.

The consideration of the hip bone as a combined ring structure allows for the assessment of osseous screw corridors in acetabular fracture surgery. In general, overlapping zones between two rings represent areas suitable for periacetabular screw fixation. For example, supraacetabular screws use the transition zone between the iliac wing ring and the acetabular ring, while infraacetabular screws (IAS) use the transition zone between the acetabular ring and the obturator ring. The superolateral part of the obturator foramen is therefore part of the infraacetabular screw corridor [4, 5]. Adaequately stable screw fixation within these rings is generally not feasible. Based on these ring considerations, Isler has summarized eight screw corridors suitable for screw fixation of acetabular fractures [6].

### The infraacetabular screw corridor (IAC)

**Fig. 2:**
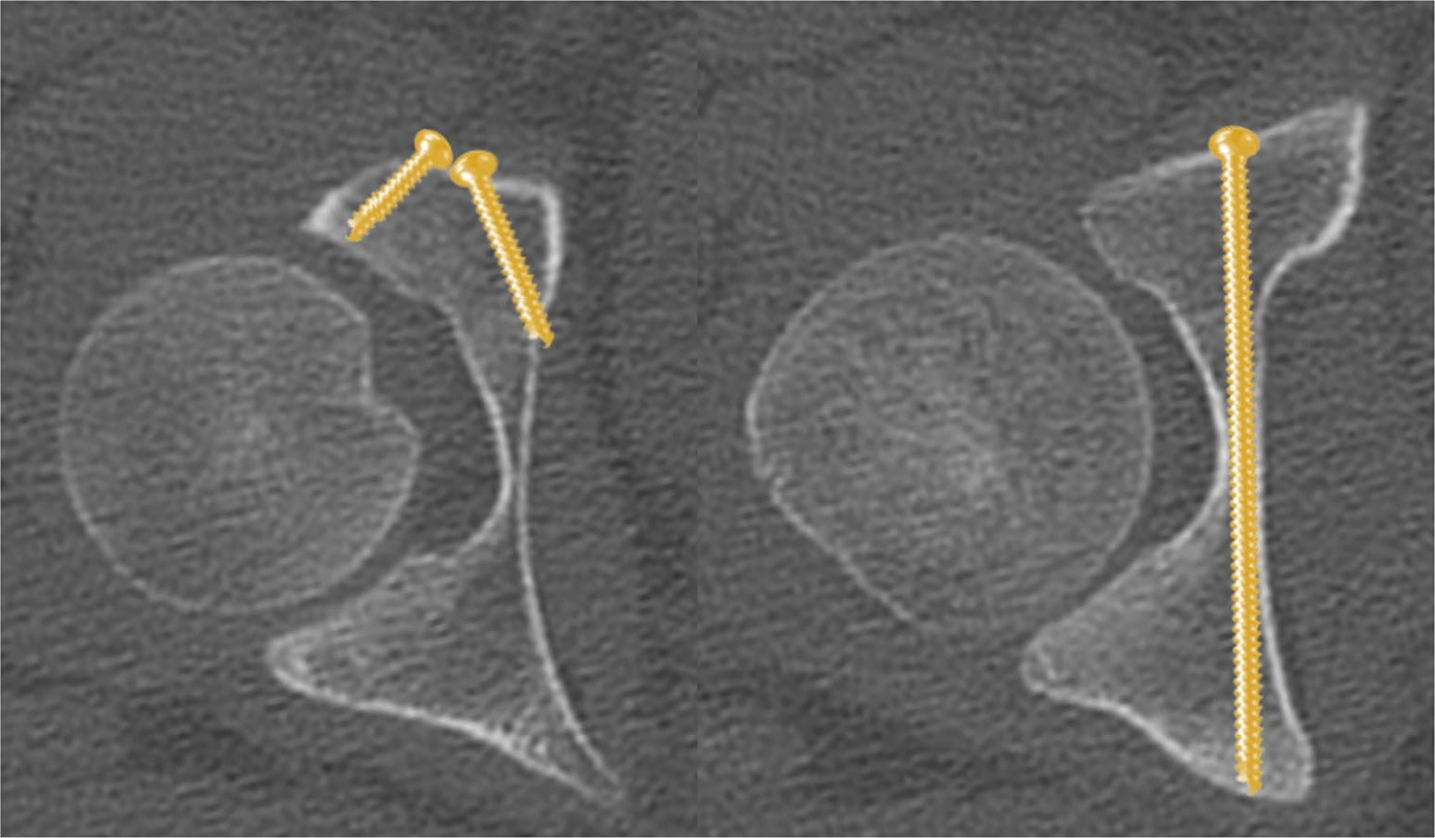
Potential screw orientations near the quadrilateral surface according to Letournel including the infraacetabular screw (right)

**Fig. 3:**
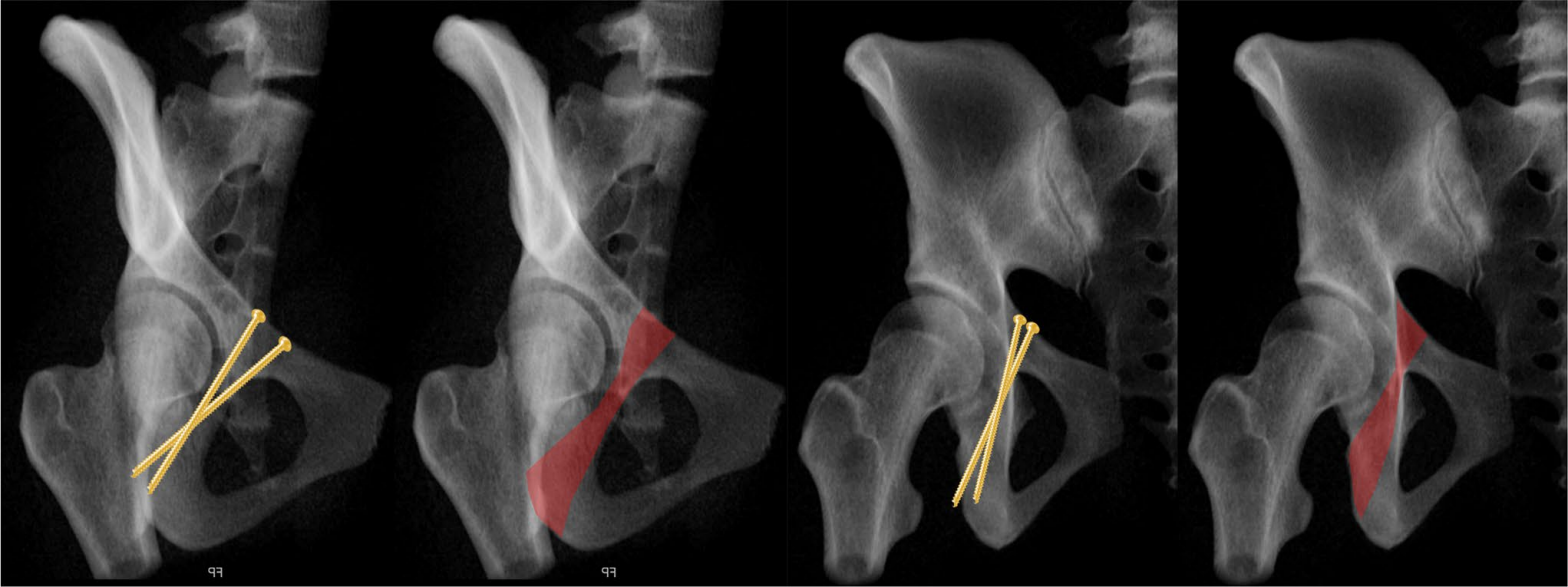
Double-cone shape of the IAC in two planes: combined obturator oblique outlet view (left) and 1/3 iliac oblique outlet view (right)

Three potential screw orientations near the quadrilateral surface were described by Letournel in the 1990s (Fig. 2 [5]), . The IAC (Fig. 2, right) is one of them and requires a screw orientation strictly parallel to the quadrilateral plate. The infaacetabular screws (IAS) may be totally embedded in the osseous floor of the acetabular fossa in the presence of a thick bony layer. Otherwise, slight thread penetration in the acetabular fossa without damaging the femoral head or the cartilage of the lunate surface may occur. Bastian described a mean distance between the screw corridor and the femoral head of 5 (1–14) mm at the level of the inferior acetabular fossa [7]. The distance depended on the lateral center edge (LCE) angle in this study. The IAC shows a double-cone shape with an isthmus located near the acetabular fovea (Fig. 3 [8]), . With an increasing diameter of the IAC a more tubular shape of the IAC was observed. The end point is located near the ischial tuberosity. The mean diameter of the IAC at the level of the isthmus was stated between 4.7 mm [9] and 7.4 mm [10]. Sex differences with larger mean diameters in men were also noted [10, 11]. Gras reported that an adequate IAC with a minimum diameter of at least 5 mm was present in 93% of all patients [10]. Zhao found rates of 94% in male and 86% in female indicating gender differences [12]. Kanezaki reported that 21.8% of the IAC in Asian people were not suitable for 3.5 mm IAS due to the anatomical shape of the IAC [13]. Yoshida decribed a rate of 68% of IAC allowing an all-inside screw trajectory [14]. In the other patients, an in-out-in screw trajectory would be feasible only.

**Fig. 4:**
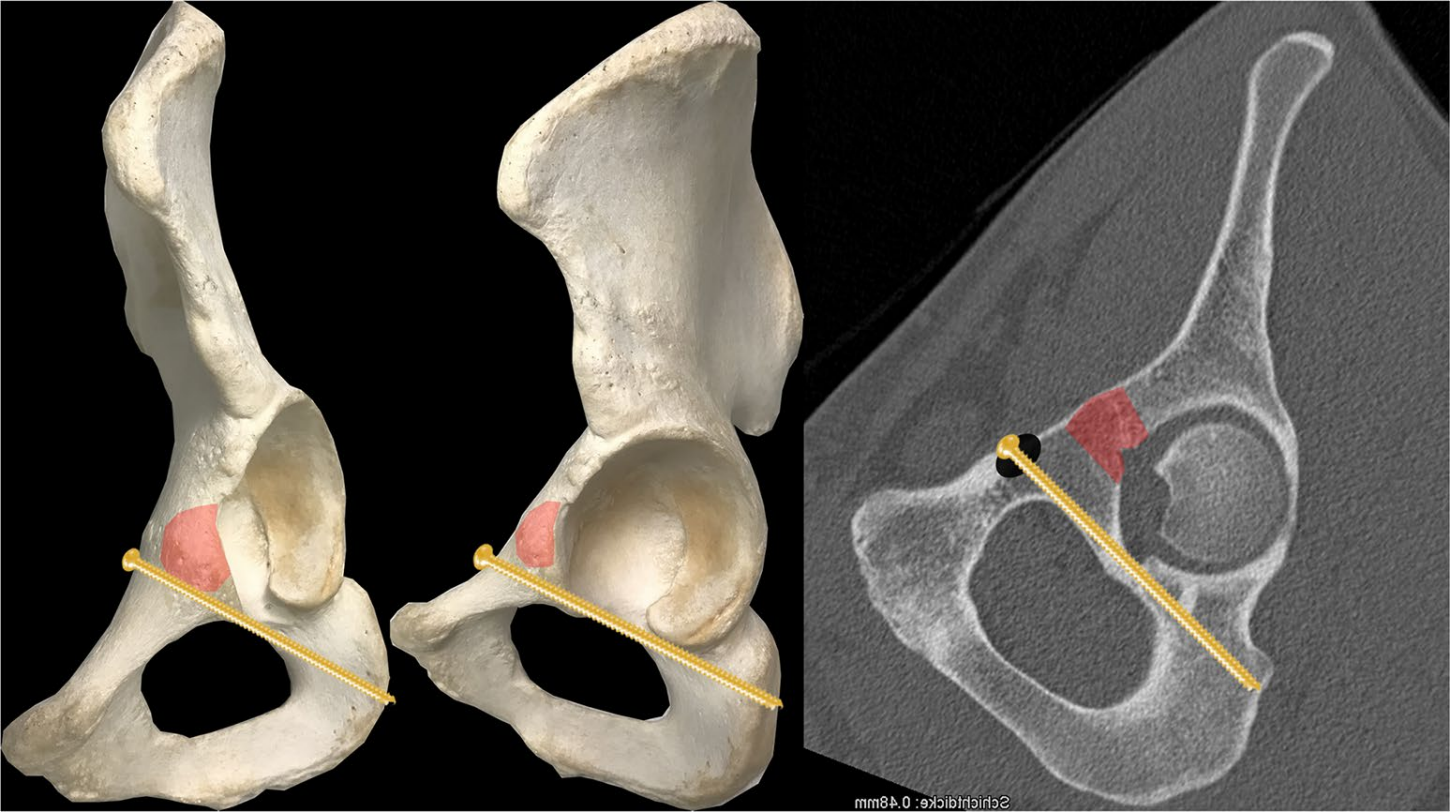
Entry-point of the infraacetabular screw approximately 1 cm caudal and 1 cm medial to the iliopectineal eminence (marked in red) according to Baumann [3]

The iliopectineal eminence (IE) is mainly used as the anatomical landmark for the intraoperative clinical assessment of the entry point of IAS. Culemann described the entry point to be located 1 cm anterior to the top of the IE in the midline of the upper pubic ramus [4]. Gras decribed the entry point to be located mediocaudal to the IE [10]. Baumann showed that the ideal entry point for the IAS was located 10 mm caudal and 10 mm medial to the iliopectineal eminence (Fig. 4 [15]), . In contrast, Kanezaki stated that the intraoperative clinical assessment of the entry point based on anatomical landmarks such as the iliopectineal eminence is of minor relevance due to the narrowness of the IAC [13]. An intraoperative radiological assessment was therefore recommended by the authors.

**Fig. 5:**
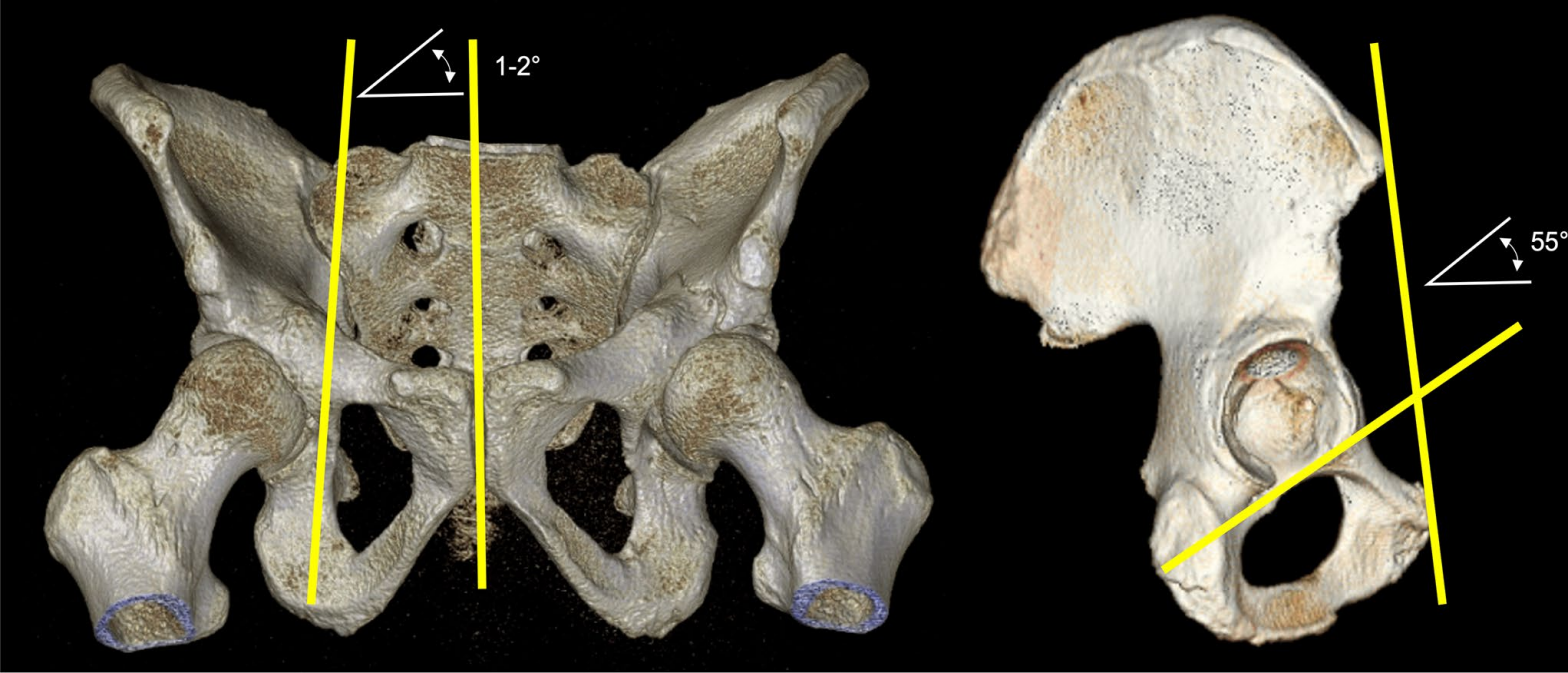
Coronal and sagittal angles of the intraoperative drill bit orientation for IAS

Screw angles for the intraoperative drill bit orientation were reported to be approximately 55° inclination on a lateral view from superoanterior to inferoposterior and 1–2° inclination on an outlet or AP view from superomedial to inferolateral (Fig. 5 [10, 11, 14]), . Slightly higher angles of up to 10° in the outlet or AP view were reported in Asian patients [14, 16].

Maximum screw lengths of IAS were found to be dependent both on the sex and the ethnicity of the patients with smaller screw length in women [14, 16] and in Asian people [12,20,31] compared to Causasians [9]. The mean maximum screw lengths were reported to be between 85 mm and 103 mm [4, 9, 10, 12–14, 16, 17].

## Radiology

Three radiographic views were recommended by Culemann for the intraoperative assessment of the entry point and the screw trajectory of IAS [18].

**Fig. 6:**
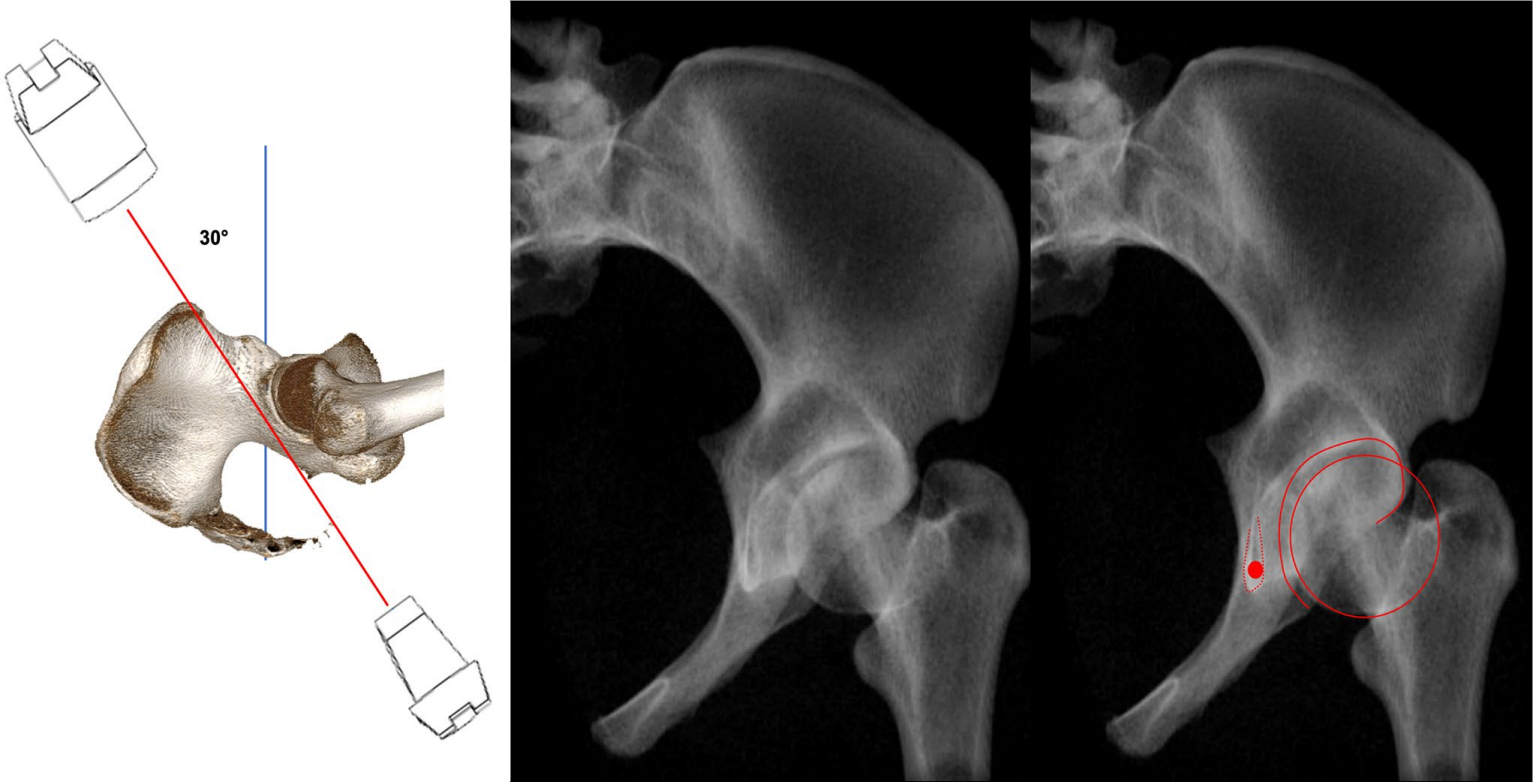
Inlet view for the identification of the entry point of IAS in the center of the teardrop figure (red dot)

**Fig. 7:**
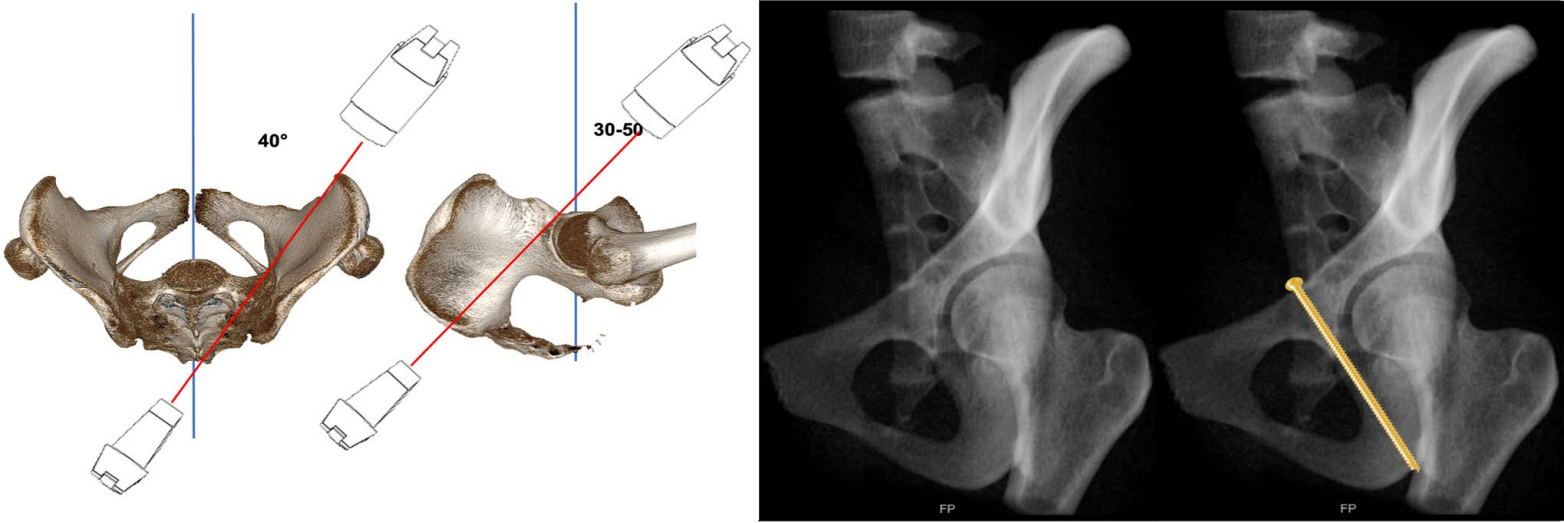
Combined Obturator Oblique Outlet (COOO) view for the assessment of the intraosseous screw trajectory

**Fig. 8:**
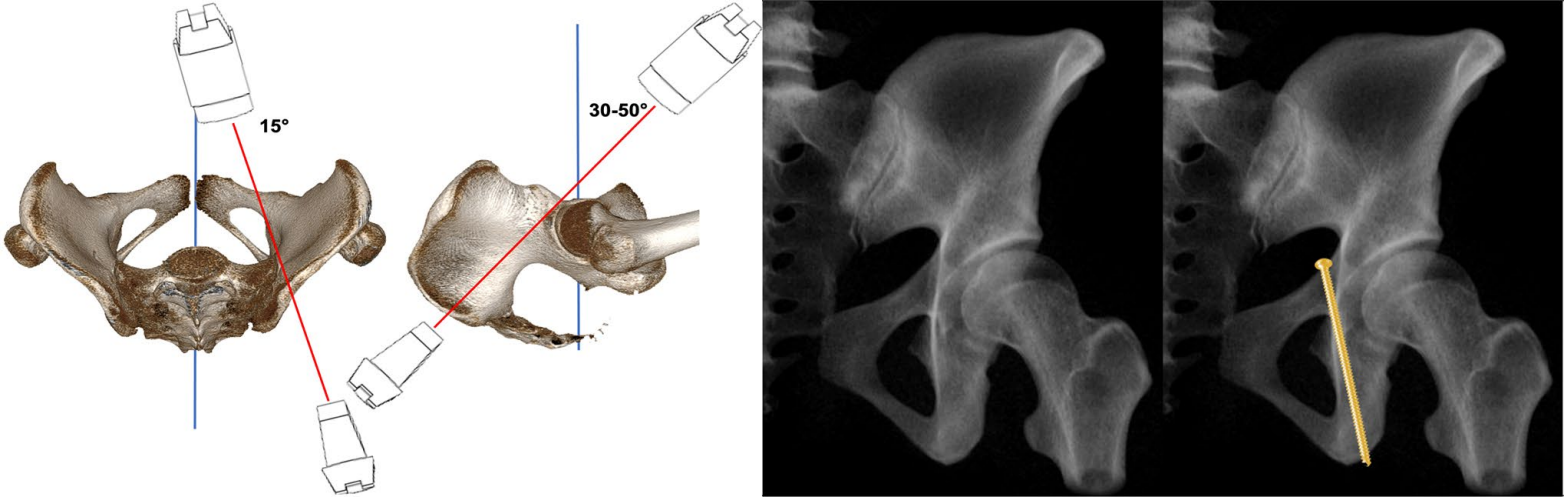
1/3 Iliac Oblique Outlet (1/3 IOO) view for the assessment of the extraarticular screw trajectory

- Inlet view (Fig. 6): The C-arm is tilted 30° cranially. The inlet view allows for the assessment of the entry point of AIS. The entry point (Fig. 6, red dot) should be in the center of the teardrop figure. The teardrop in the inlet view is formed medially by the quadrilateral plate, laterally by the acetabular fossa, and inferiorly by the acetabular notch [19].
- Combined Obturator Oblique Outlet view (COOO, Fig. 7): The C-arm is rotated approximetaly 40° to the fracture side with an additional 30°-50° caudal tilting of the C-arm. The COOO view allows for the assessment of the intraosseous screw trajectory mainly at the superior border of the obturator ring.
- 1/3 Iliac Oblique Outlet view (1/3 IOO, Fig. 8): The C-arm is rotated approximately 15° to the uninjured side with an additional 30°-50° caudal tilting of the C-arm. The 1/3 IOO allows for the assessment of the extraarticular screw positioning.

Lu confirmed the assessment of the optimal entry point within the teardrop figure in the inlet plane [11]. Other authors, however, recommended different views for the radiological assessment of IAS. Gras, for example, recommended the COOO, a ½ IOO and an AP view of the pelvis [20]. Lim found that the outlet view allowed for the assessment of the screw trajectory in approximately 90° of the cases [19]. In general, Elmhiregh showed that the probabilty of an intraarticular screw trajectory in a concave surface such as the acetabulum is very low, if one particular view shows a distinct extraarticular trajectory [21].

## Biomechanics

**Fig. 9:**
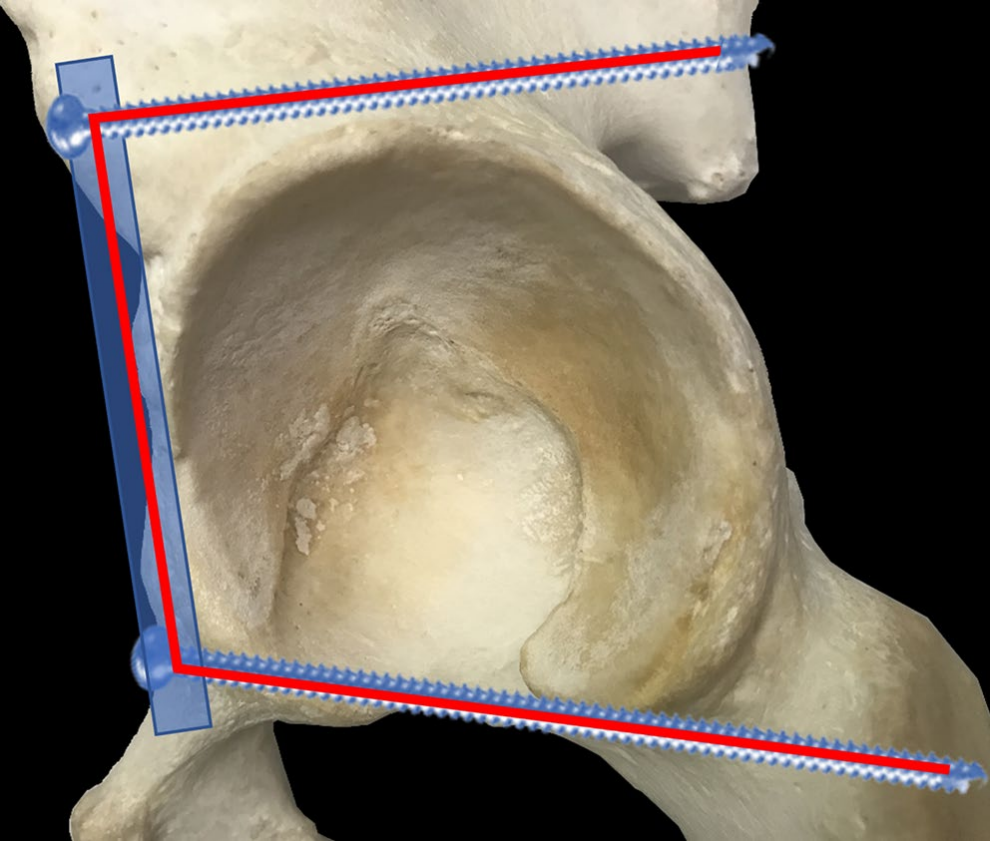
Concept of the „periacetabular fixation frame“ consisting of a supraacetabular screw, an infraacetabular screw and a suprapectineal plate

From a biomechanical point of view, infraacetabular screws should allow for an increased stability of the acetabular fixation by closing the periacetabular fixation frame without requiring an additional posterior approach (Fig. 9). A periacetabular fixation frame consists of a supraacetabular screw, an infraacetabular screw and a suprapectineal plate. Accordingly, several biomechanical studies confirmed this concept.

Culemann performed a biomechanical study using a model of anterior column and posterior hemitransverse (ACPHT) fractures in a single leg stance model [22]. It was shown that a suprapectineal plate combined with long periarticular screws, i.e. supra- and infracetabular screws, showed the highest stiffness by closing the periacetabular fixation frame. These findings were confirmed in a similar study by Spitler in a model simulating a standing position [23] and in a study by Chen [24]. Marintschev performed a biomechanical study applying a model of high anterior column (AC) fractures in a single leg stance model [25]. Different non-locking and locking suprapectineal plates were tested with and without IAS. Additional IAS increased the stiffness of the fixation constructs by up to 50%, while locking plates had no signficant effect. These findings were confirmed in similar biomechanical study by Gras [26]. Graul compared a standard suprapectineal plate with an anatomically pre-shaped suprapectineal plate with integration of an internal posterior column part [27]. The anatomically pre-shaped suprapectineal plate provided higher fixation stiffness than the standard plate. Adding an IAS to the standard plate resulted in comparable stiffness to the preshaped plate, while adding an IAS to this plate did not further increase the stiffness of the construct.

In contrast, Hinz found in a biomechanical study using a model of anterior column and posterior hemitransverse (ACPHT) fractures in a single leg stance model that suprapectineal plate fixation with an additional posterior column screw [28] showed higher fixation stiffness than a suprapectineal plate fixation with an additional IAS [29]. IAS do not cross the transverse posterior hemitransverse fracture, while posterior column screws cross this fracture component nearly perpendicular and may therefore act as lag screws.

## Clinics

IAS have gained increasing interest and are used more and more often in the last decade. The available clinical studies, however, mainly focus on the safety of the intraoperative application of IAS using different modalities. Studies showing the additional clinical value of IAS, however, are still missing.

Gras reported in 2012 the use of a 3D navigation system for the insertion of IAS [20]. Cao compared robotic-assisted versus free-hand insertion of IAS. Robotic-assistance resulted in less surgical time (14.4 min vs. 26.3 min). There were, however, no differences in the clinical follow-up [30]. Pagano compared navigation-assisted application versus conventional application of IAS with no clinical differences between the study groups during the follow-up [9].

## Conclusion

In conclusion, several biomechanical studies have shown that the additional use of IAS resulted in an increase fixation stiffness in AC and ACPHT fractures by closing the periacetabular fixation frame without requiring an additional posterior approach. Clinical studies, however, mainly focused on the safety of the screw insertion. The scientific evidence for an improved clinical outcome following the use of IAS is still missing.
